# Parkinsonism Is Associated with Altered SMA-Basal Ganglia Structural and Functional Connectivity in Frontotemporal Degeneration

**DOI:** 10.3390/biomedicines11020522

**Published:** 2023-02-10

**Authors:** Claudia Piervincenzi, Antonio Suppa, Nikolaos Petsas, Andrea Fabbrini, Alessandro Trebbastoni, Francesco Asci, Costanza Giannì, Alfredo Berardelli, Patrizia Pantano

**Affiliations:** 1 Department of Human Neurosciences, Sapienza University of Rome, 00185 Rome, Italy; 2 IRCCS NEUROMED, 86077 Pozzilli, Italy

**Keywords:** frontotemporal degeneration (FTD), parkinsonism, supplementary motor area (SMA), probabilistic tractography, resting-state functional MRI

## Abstract

Background: Patients with frontotemporal degeneration (FTD) often manifest parkinsonism, which likely results from cortical and subcortical degeneration of brain structures involved in motor control. We used a multimodal magnetic resonance imaging (MRI) approach to investigate possible structural and/or functional alterations in FTD patients with and without parkinsonism (Park+ and Park−). Methods: Thirty FTD patients (12 Park+, 18 Park−) and 30 healthy controls were enrolled and underwent 3T MRI scanning. MRI analyses included: (1) surface-based morphometry; (2) basal ganglia and thalamic volumetry; (3) diffusion-based probabilistic tractography of fiber tracts connecting the supplementary motor area (SMA) and primary motor cortex (M1) to the putamen, globus pallidus, and thalamus; and (4) resting-state functional connectivity (RSFC) between the aforementioned regions. Results: Patients in Park+ and Park− groups showed comparable patterns of cortical thinning in frontotemporal regions and reduced thalamic volume with respect to controls. Only Park+ patients showed reduced putaminal volume and reduced fractional anisotropy of the fibers connecting the SMA to the globus pallidus, putamen, and thalamus, with respect to controls. Park+ patients also showed decreased RSFC between the SMA and putamen with respect to both Park− patients and controls. Conclusions: The present findings support the hypothesis that FTD patients with parkinsonism are characterized by neurodegenerative processes in specific corticobasal ganglia-thalamocortical motor loops.

## 1. Introduction

Frontotemporal degeneration (FTD) refers to a group of neurodegenerative disorders leading to early-onset dementia. FTD is clinically characterized by a variable association of cognitive impairment, behavioral signs, and language disorders [1,2]. According to the prominent clinical features present, FTD is classified as either a behavioral variant (bv-FTD) or a language variant. The language variant is referred to as primary progressive aphasia (PPA), which can be further subdivided into non-fluent (nfv-PPA), semantic (sv-PPA), and logopenic (lv-PPA) variants [3].

About 20–40% of FTD patients also manifest clinical signs of parkinsonism [4,5,6,7]. Parkinsonian signs are more frequently observed in bv-FTD and nfv-PPA patients as compared with sv-PPA patients [6]. The pathophysiology of parkinsonism in patients with FTD has been poorly investigated to date [8,9,10]. Several neuroimaging studies have demonstrated structural, functional, and metabolic changes in subcortical structures, including the basal ganglia, in patients with FTD [5,11,12,13,14], though none have specifically compared patients with and without parkinsonism to better understand the pathophysiology of parkinsonism in FTD.

The present study aimed to investigate pathophysiological mechanisms underlying parkinsonian signs in patients with FTD. For this purpose, we examined possible structural and functional changes of the corticobasal ganglia-thalamo-motor cortex circuit in FTD patients with (Park+) or without parkinsonism (Park−), by using a multimodal magnetic resonance imaging (MRI) approach that included surface-based morphometry, basal ganglia and thalamic volumetry, diffusion-based probabilistic tractography, and resting-state functional connectivity (RSFC). The choice of investigating such network follows the experimental evidence that structural and functional changes in the corticobasal ganglia-thalamo-motor cortex circuit are the key pathophysiological underpinning of parkinsonism [15,16,17,18,19,20].

Our hypothesis is that FTD patients manifesting parkinsonism would be characterized by prominent structural and functional changes in critical nodes of the corticobasal ganglia-thalamo-motor cortex circuit, such as the supplementary motor area (SMA) and primary motor cortex (M1), putamen, globus pallidus, as well as in the thalamus.

## 2. Materials and Methods

### 2.1. Participants

Participants were recruited from the Department of Human Neurosciences, Sapienza University of Rome, Italy, and consisted of 30 patients (21 M, mean age ± standard deviation (SD): 70 ± 7 years) with a clinically probable diagnosis of FTD [21,22]. The FTD study cohort included 12 Park+ patients (10 M, mean age ± SD: 73 ± 6 years) and 18 Park− patients (11 M, mean age ± SD: 68 ± 8 years). None of them took medications with potential drug-induced parkinsonism effects, such as typical and atypical antipsychotics, dopamine depleters, or antiemetics. The study also included 30 age- and sex-matched healthy subjects (21 M, mean age ± SD: 68 ± 6 years). All participants were right-handed and native Italian speakers. The diagnosis of probable FTD and its categorization as bv-FTD or nfv-PPA was based on recent international consensus criteria [21,22]. The clinical diagnosis of probable FTD was also confirmed in follow-up clinical evaluations [21,22]. The diagnosis was imaging-supported since, in addition to conventional MRI, all patients also underwent fluorodeoxyglucose positron emission tomography (FDG-PET), which revealed hypometabolic patterns consistent with a diagnosis of FTD and the respective clinical variant [21,22,23]. Clinical and instrumental (i.e., electromyography) investigations excluded upper and lower motor neuron involvement.

None of the patients with parkinsonism showed a clinical response to levodopa administration. Demographic and clinical features of included patients are provided in Table 1.

### 2.2. Clinical and Neuropsychological Assessment

We used the most recent clinical criteria for the diagnosis of parkinsonism [24], which was rated by means of the Movement Disorder Society-sponsored revision of the Unified Parkinson’s Disease Rating Scale part III (MDS-UPDRS-III) [25]. In the present group of patients, parkinsonism included bradykinesia, rigidity, parkinsonian gait, and postural instability. In addition, tremor was less frequently present. Patients underwent a neuropsychological evaluation including the Mini-Mental State Examination (MMSE) [26], frontal assessment battery (FAB) [27], and trail making test (TMT) (subtypes A and B) [28]. Language function was assessed in all patients using verbal semantic fluency (VSF) [29,30] and verbal phonemic fluency (VPF) tests [29]. Dementia severity was also assessed using the Clinical Dementia Rating scale & Frontotemporal Dementia (CDR-FTD) [31].

### 2.3. MRI Data Acquisition

Images were acquired with a 3-Tesla (3T) scanner (Siemens Magnetom Verio, Erlangen, Germany) and a 12-channel head coil designed for parallel imaging (GRAPPA). The following sequences were acquired:

- High-resolution three-dimensional (3D) T1-weighted (T1-3D) MPRAGE sequence (repetition time (TR) = 2400 ms, echo time (TE) = 2.12 ms, inversion time (TI) = 1000 ms, flip angle = 8°, field of view (FOV) = 256 mm, matrix = 256 × 256, 176 sagittal slices 1-mm thick, no gap);
- Diffusion tensor imaging (DTI) single-shot, echo-planar, spin-echo sequence with 10 interspersed volumes of b = 0 (b0) and 64 gradient directions, TR = 4600 ms, TE = 78 ms, multiband acceleration factor = 2, monopolar diffusion scheme, FOV = 192 mm, matrix = 96 × 96, b = 1000 s/mm^2^, 72 contiguous axial 2-mm thick slices;
- Blood oxygen level-dependent (BOLD) single-shot echo-planar imaging (TR = 3000 ms, TE = 30 ms, flip angle = 89°, FOV = 192 mm, 64 × 64 matrix, 50 contiguous axial 3-mm thick slices, 140 volumes, voxel size = 3 mm^3^), with all patients instructed to close their eyes and remain awake during the resting-state functional MRI acquisitions;
- Dual turbo spin-echo, proton density (PD) and T2-weighted images (TR = 3320 ms, TE1 = 10 ms, TE2 = 103 ms, FOV = 220 mm, matrix = 384 × 384, 25 axial slices 4-mm thick, 30% gap);
- High-resolution 3D fluid-attenuated inversion recovery (FLAIR) sequence (TR = 6000 ms, TE = 395 ms, TI = 2100 ms, FOV = 256 mm, matrix = 256 × 256, 176 sagittal slices 1-mm thick, no gap).
- Two expert radiologists (PP and CG, with more than 20 and 10 years of experience, respectively) examined all MRIs to assess the presence of T2 and T2 FLAIR white matter hyperintensities (WMH). The amount of WMH was quantified using the four-stage Fazekas visual rating scale (Fazekas 0–1 = no to mild WMH, Fazekas 2 = moderate WMH, Fazekas 3 = severe WMH) [32].

### 2.4. MRI Analysis

#### 2.4.1. Data Preprocessing

Anatomical and functional preprocessing was performed using fMRIPrep 20.1.1 [33,34]; RRID:SCR_016216, which is based on Nipype 1.5.0 [35,36]; RRID:SCR_002502. Diffusion data were preprocessed using different FDT tools (FMRIB Diffusion Toolbox, part of FSL (FMRIB’s Software Library v.6.0.4, http://www.fmrib.ox.ac.uk/fsl/ [37], accessed on 1 May 2021). For a description of anatomical, functional, and diffusion preprocessing, please see the Supplementary Materials.

#### 2.4.2. Cortical Thickness

Entire cortex analyses were computed to explore cortical thickness in Park−, Park+, and healthy controls. Statistical maps were obtained using FreeSurfer’s Query, Design, Estimate, Contrast (QDEC) interface. First, each patient group was compared with the control group, followed by the comparison of the two clinical groups with each other. For each hemisphere, the general linear model was computed vertex-wise to analyze cortical thickness, accounting for the effects of age and sex. For Park+ patients, correlation analyses were also performed to assess possible correlations between MDS-UPDRS-III scores and cortical thickness. Cortical maps were smoothed using a 10-mm full-width at half maximum Gaussian kernel and the results were visualized by overlaying significant cortical areas onto semi-inflated cortical surfaces. Multiple comparisons were corrected with Monte Carlo simulation using a *p*-value set at <0.05.

#### 2.4.3. Basal Ganglia and Thalamus Volumetry

Left and right putaminal, pallidal, and thalamic volumes were extracted directly from FreeSurfer’s final output and normalized for head size using the total intracranial volume, which was also obtained directly from FreeSurfer, and used for statistical analyses.

#### 2.4.4. Selection of Regions of Interest (ROIs)

Since the main aim of the study was to better understand the pathophysiology of parkinsonism in FTD patients, we selected the most important cortical and subcortical areas involved in motor circuit. We chose to investigate structural and functional connectivity between two cortical regions (the SMA and M1) and three subcortical regions (the putamen, globus pallidus, and thalamus). The putamen and globus pallidus were chosen since they are the main basal ganglia input and output structures, respectively. The thalamus was chosen since it serves as a relay structure between the basal ganglia, cerebellum, and cortex. Direct cortico-pallidal connectivity was investigated due to experimental evidence demonstrating direct projections from the cerebral cortex to the globus pallidus in animals [38,39,40] and more recent evidence that used diffusion tractography to demonstrate direct cortico-pallidal projections in humans [41,42,43].

Cortical ROIs were selected from probabilistic atlases: M1 (upper limb region) was taken from the Brainnetome atlas (https://atlas.brainnetome.org/download.html, accessed on 1 May 2021) [44], and the Juxtapositional lobule cortex (formerly the SMA) was selected from the Harvard-Oxford Cortical Structural Atlas (http://www.fmrib.ox.ac.uk/fsl/data/atlasdescriptions.html, accessed on 1 May 2021). Both M1 and SMA ROIs were thresholded at 25% and divided on the sagittal plane *x* = 0 into right and left regions. Subcortical ROIs were obtained from FreeSurfer-derived segmentations: right and left globi pallidi, putamina, and thalami. Cortical and subcortical regions were transformed from standard and structural space, respectively, into diffusion space using previously generated registrations.

#### 2.4.5. Structural Connectivity—Tractography

Probabilistic tractography was performed within each participant’s diffusion space using BedpostX [45] with default parameters. Streamline probability distribution maps were generated from each cortical region (SMA, M1) to each subcortical target structure (left and right globi pallidi, putamina, and thalami). In each map, the cortical region was specified as a seed, the subcortical region as a target, and the contralateral hemisphere as an exclusion mask. The subcortical target region was also specified as a termination mask to define the exclusive and exact connections between a given seed and target [46]. Pathway probability maps were normalized for seed size by dividing the probability maps by the total number of successfully generated streamlines, and spurious connections were removed by thresholding the resulting maps by 5% [46,47]. Thresholded probability maps were then binarized and overlaid on fractional anisotropy (FA) maps from which average values were extracted [13]. FA quantified the degree of anisotropic diffusion within the single voxel; higher FA values are thought to reflect better WM integrity as a result of greater intravoxel coherence of fiber orientation, axon density, and diameter and/or myelination [48,49].

#### 2.4.6. Functional Connectivity—ROI-to-ROI Correlation Analyses

For each cortical and subcortical ROI, individual mean time courses were obtained. Pearson’s correlation coefficient (Fisher z–transformed) was used to estimate the strength of the functional connection between each cortical ROI (SMA, M1) with the three subcortical ROIs within each hemisphere.

### 2.5. Statistical Analyses

Statistical analyses were performed using SPSS statistics software (version 22.0). Between-group differences (all patients vs. controls and Park+ vs. Park− patients) in demographic, clinical, and neuropsychological parameters were tested using the Mann–Whitney U and Chi-square tests for continuous and dichotomous variables, respectively (*p* < 0.05 for null hypothesis rejection). The Kruskal–Wallis non-parametric test was used to investigate differences between groups (Park+, Park−, and controls) in left and right global cortical thickness and cortical, basal ganglia, and thalamic volume. Regarding structural and functional connectivity analyses, since no significant differences in right-left FA or RSFC values were detected (Wilcoxon signed-rank test), we averaged the right and left values and performed the Kruskal–Wallis test to investigate between-group differences in FA values of the SMA-subcortical and M1-subcortical WM tracts and RSFC values of the SMA-subcortical and M1-subcortical ROI pairs. All results were Bonferroni corrected (corrected alpha level = 0.017). For Park+ patients, Spearman’s rank correlation test was used to assess correlations between MDS-UPDRS-III scores and cortical measures, basal ganglia and thalamic volume, FA, and RSFC measures.

## 3. Results

Demographic and clinical characteristics of Park+, Park−, and controls are reported in Table 1. There were no significant differences in terms of age or sex distribution between patients and controls and between Park+ and Park− patients. No significant differences were found in clinical and neuropsychological measures between Park+ and Park− patients. The mean MDS-UPDRS-III score in the Park+ patient group was 17.8 (SD 10.4). 18 FTD patients were graded as Fazekas 0, 8 patients were Fazekas 1 (4 Park+ and 4 Park−), and 4 patients were Fazekas 2 (2 Park+ and 2 Park−). No patients showed T2 and T2 FLAIR WMH in brain regions critical for vascular parkinsonism [50].

### 3.1. Cortical Thickness and Basal Ganglia/Thalamic Volumetry

The Kruskal–Wallis test showed significant between-group differences for left and right global cortical thickness and cortical volume; post hoc tests indicated that both Park+ and Park− patients had reduced global cortical thickness and volume with respect to controls whereas Park+ and Park− patients showed comparable values (Table 2).

**Figure 1:**
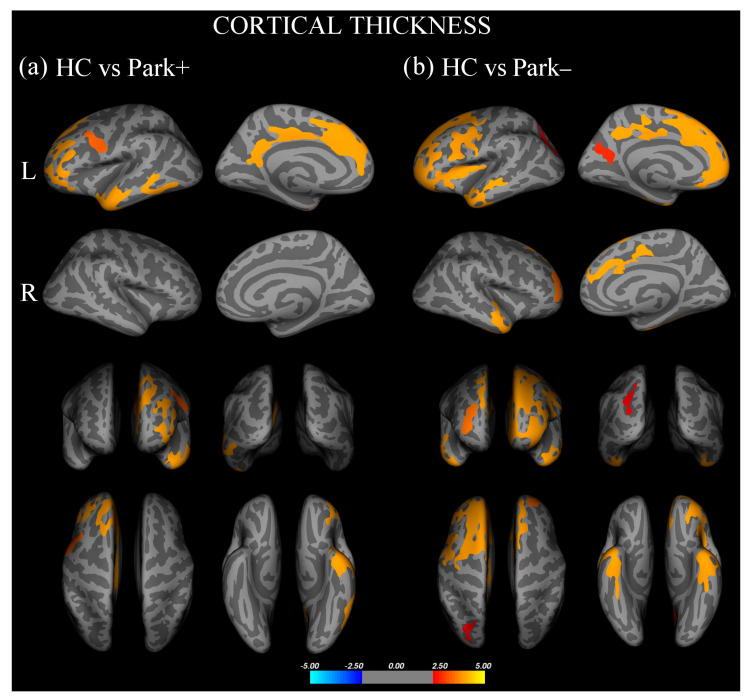
Cortical areas showing significant thinning in (**a**) FTD patients with parkinsonism (Park+) compared to healthy controls (HC) and (**b**) FTD patients without parkinsonism (Park−) compared with HC.Results are displayed on QDEC’s semi-inflated cortical surfaces. Top row: left (L) lateral and L medial. Second row: right (R) lateral and R medial. Third row: anterior and posterior views. Bottom row: superior and inferior views. The color bar indicates the cluster significance level (−log10 (*p*-value)).

Vertex-wise analysis of cortical thickness showed that, with respect to controls, Park+ patients presented significant thinning in the left inferior frontal gyrus, pars orbitalis, rostral middle frontal and superior frontal gyri, and inferior temporal and precentral gyri (Figure 1a, Supplementary Table S1). With respect to controls, Park− patients showed significant thinning of the left inferior frontal gyrus, pars opercularis, bilateral rostral middle frontal gyri, right superior frontal gyrus, bilateral inferior temporal gyri, left precuneus, and superior parietal and fusiform gyri (Figure 1b, Supplementary Table S1). No significant difference in cortical thickness was found between Park+ and Park− patients.

Regarding subcortical volumes, the Kruskal–Wallis test showed significant between-group differences for left and right putamina and thalami; post hoc tests showed that, with respect to controls, Park+ patients presented significantly lower putaminal and thalamic volumes bilaterally, while Park− patients only showed significantly lower left thalamic volume (Table 2).

### 3.2. Structural Connectivity

**Figure 2:**
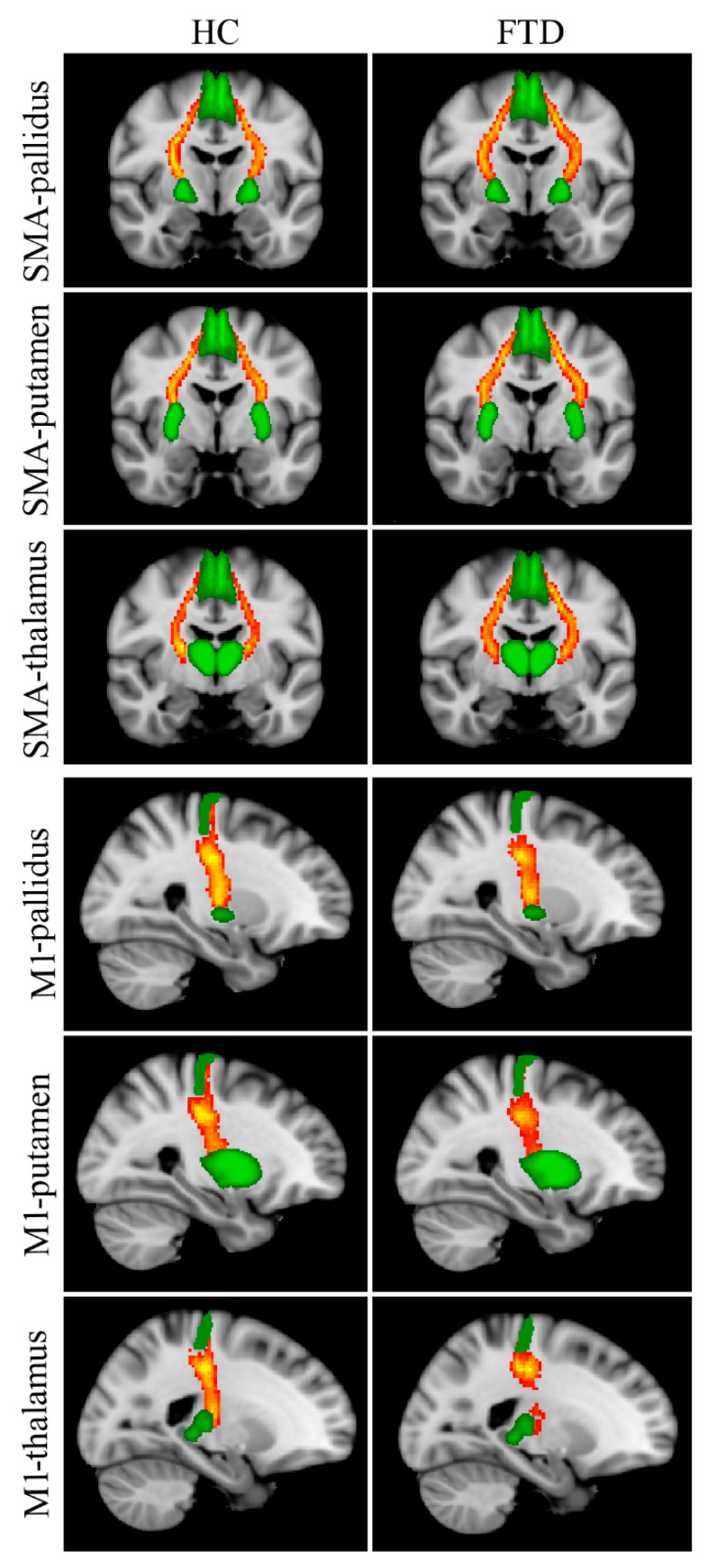
Reconstructed white matter pathways in healthy controls (HC) (**left panel**) and all FTD patients (Park+ and Park−, **right panel**) overlaid onto the MNI152 standard brain. Red-yellow colors reflect the extent of spatial overlap of reconstructed tracts between participants, with red indicating at least 40% overlap and yellow indicating 100%. Green areas represent regions of interest used for probabilistic tractography. Differences in fractional anisotropy (FA) values between controls and FTD patients with and without parkinsonism are reported in Table 3.

Reconstructed WM tracts, i.e., SMA-pallidus, SMA-putamen, SMA-thalamus, M1-pallidus, M1-putamen, M1-thalamus, are shown in Figure 2. The Kruskal–Wallis test revealed significant between-group FA differences in all tracts connecting the SMA with subcortical nuclei (Table 3); post hoc tests showed significantly lower FA values in Park+ patients with respect to controls for each tract. No significant differences were found between Park− patients and the other two groups. Between-group differences in FA for M1-pallidus, M1-putamen, and M1-thalamus did not survive Bonferroni correction.

### 3.3. Functional Connectivity

The Kruskal–Wallis test showed significant between-group RSFC differences for the SMA-putamen ROI pair; post hoc tests showed significantly lower RSFC values in Park+ patients with respect to both controls and Park− patients (Table 3). Differences in RSFC for the remaining ROI pairs did not survive Bonferroni correction.

### 3.4. Correlation Analyses

No significant correlations were found between MDS-UPDRS-III scores and any MRI measure.

## 4. Discussion

The present study was aimed at investigating the pathophysiological mechanisms underlying parkinsonian signs in patients with FTD by examining possible structural and functional changes in the basal ganglia-thalamo-motor cortex circuit. We found comparable patterns of cortical thinning in frontal and anterior temporal regions, and reduced thalamic volume in FTD patients with and without parkinsonism with respect to controls. More interestingly, we found that only patients with parkinsonism showed reduced putaminal volume, prominent loss of WM microstructural integrity of the fibers connecting the globus pallidus, putamen, and thalamus with the SMA, and decreased RSFC between the SMA and putamen. The results suggest that structural and functional alterations in critical nodes of the corticobasal ganglia-thalamo-cortical motor loop differentiate FTD patients with parkinsonism from FTD patients who do not manifest motor symptoms. These neuroimaging findings provide further insight into the pathophysiological underpinning of parkinsonism in FTD.

### 4.1. Cortical Thickness in FTD

In the present study, all FTD patients were characterized by significant cortical thinning in frontal and anterior temporal regions, a finding in line with previous observations [51,52]. An additional finding was that the amount of structural changes in cortical GM was comparable in Park+ and Park− patients. This finding suggests that parkinsonism in FTD cannot be attributed to prominent GM loss in these cortical regions. Finally, cortical thinning was mainly found in the left hemisphere, which can be explained by the higher number of FTD patients with the nfv-PPA variant in the present study (19 of 30 patients) and is in line with previous findings of cortical thinning in the language variants of frontotemporal lobar degeneration [53].

### 4.2. Striatal Degeneration in FTD with Parkinsonism

A relevant finding of this study is the prominent putaminal atrophy in Park+ patients as compared with controls. The putamen is the striatal component of the corticobasal ganglia-thalamocortical motor loop. Since dopaminergic denervation of the putamen is the main pathophysiological correlate of parkinsonian signs in patients with Parkinson’s disease (PD), striatal degeneration would also represent a key pathophysiological mechanism of parkinsonism in FTD. Although atrophy of the putamen has previously been reported in all FTD clinical variants [54,55,56,57], no study has directly associated putaminal atrophy with parkinsonian signs [12]. The present results are consistent with a previous ante-mortem MRI study in neuropathologically-confirmed FTD that showed an association between putaminal volumetric loss and parkinsonian symptoms in patients with tau- and ubiquitin-predominant intracellular inclusions [11]. The present results suggest that striatal dopamine deficiency is the key pathophysiological mechanism underlying parkinsonism in FTD patients, in line with previous positron emission tomography (PET) and single-photon emission computerized tomography (SPECT) studies in FTD, showing loss of dopaminergic neurons in the caudate and the putamen [5,58].

Another finding of the study was that the amount of atrophy in the thalamus was comparable in Park+ and Park− patients and controls. The observation of thalamic atrophy is in line with previous studies in FTD [14,59] and supports the conclusion that thalamic atrophy is unlikely to represent a major contribution to the pathophysiology of parkinsonism in FTD.

### 4.3. Reduced SMA-Basal Ganglia Structural and Functional Connectivity in FTD with Parkinsonism

A further finding of this study was that FTD patients, particularly those with parkinsonism, were characterized by a loss of WM integrity in the structural connectivity between the SMA and specific subcortical structures, such as the putamen and globus pallidus. The alteration of WM integrity of tracts connecting the SMA to the basal ganglia and thalamus is consistent with the findings of a recent study showing reduced WM integrity of fiber tracts connecting the striatum and thalamus to the prefrontal cortex in bv-FTD patients [13]. In that study, the authors supported the hypothesis of deficient top-down modulation, which may explain deficits in executive functioning, social cognition, and behavioral alterations in bv-FTD patients. In the present study, we found evidence that parkinsonism in FTD is associated with WM integrity loss in key structural connections between nodes of the corticobasal ganglia-thalamo-cortical motor loop, such as SMA-to-basal ganglia projections. It is known that in PD patients, most parkinsonian signs and symptoms reflect a principal impairment in medial cortical areas [15,16,17,18]. A similar argument would also explain why we failed to find significant structural WM changes in fibers connecting the basal ganglia/thalamus and lateral cortical regions, such as M1, in our cohort of FTD patients with and without parkinsonism compared with controls. We cannot exclude that WM integrity loss would occur also in lateral cortical regions, such as M1, in patients with clinically evident signs of upper motor neuron syndrome. However, patients with clinical signs of cortico-spinal degeneration were not included in the present study. In conclusion, we suggest that the altered structural connectivity between the SMA and basal ganglia observed in Park+ patients is a relevant pathophysiological mechanism in patients with FTD and parkinsonism.

Concerning functional connectivity results, we found a significant reduction in RSFC between the SMA and putamen in Park+ patients. This finding is consistent with tractography results and further supports the hypothesis that impaired connectivity between the SMA and putamen is relevant in the pathophysiology of parkinsonism in FTD. Our findings are also consistent with previous functional studies showing reduced functional connectivity between the SMA and putamen in patients with PD [19,20]. We therefore conclude that our observations point to abnormal structural and functional connectivity between the SMA and basal ganglia as a relevant pathophysiological mechanism responsible for parkinsonism in patients with FTD.

### 4.4. Study Strengths and Limitations

The strengths of our study are that firstly, we focused for the first time on parkinsonism, a common but understudied feature in FTD. Secondly, we used a multimodal MRI approach including well-established image analysis techniques to compare structural and functional differences between FTD patients with and without parkinsonism and age- and sex-matched healthy controls. Thirdly, by including only FTD patients who were not taking atypical antipsychotics, we tried to address the possible confounding role of neuroleptics (drug-induced parkinsonism). In the present study, Park+ patients showed specific structural and functional alterations, which might suggest that the role of antipsychotics is not causative and that these drugs can only facilitate the emergence of parkinsonian signs in FTD patients.

The main limitations of the present work are the relatively small number of patients enrolled and the heterogeneous clinical variants that characterize FTD. The small number of patients with parkinsonism (N = 12) may also explain the lack of significant correlations between MDS-UPDRS scores and structural/functional alterations. Concerning the clinical variants included in the study, we found that clinically overt parkinsonism was present in 40% of recruited patients (12 out of 30), a percentage consistent with previous observations in FTD cohorts [6,60]. However, we observed that the majority of our patients with parkinsonism had the nfv-PPA variant (*n* = 9) rather than bv-FTD, the variant most commonly associated with parkinsonism [4,61]. Despite the difficulty of recruiting FTD patients who were not taking medications with potential drug-induced parkinsonism effects, further research on larger samples will be critical to better characterize the effect of neurodegenerative processes in specific corticobasal ganglia-thalamocortical motor loops in FTD patients with parkinsonism.

Furthermore, although our findings point to dopaminergic deficit in patients with FTD, the lack of response to L-Dopa we observed in our cohort would suggest a post-synaptic disorder (i.e., putaminal atrophy) rather than a pre-synaptic component secondary to nigrostriatal degeneration that has been also reported in FTD [5,58]. However, since our FTD patients did not undergo dopamine transporter scintigraphy, a pre-synaptic deficit cannot be fully ruled out. Finally, none of the patients had typical clinical features and disease progression suggesting a diagnosis of Alzheimer’s disease and corticobasal degeneration making it, therefore, unlikely that some of the patients we enrolled had these pathological conditions. Regarding methodology, although tractography offers enormous potential for the study of brain connectivity, it also suffers from well-known limitations [62], which may be partially overcome by the multimodal MRI approach we used. The agreement between diffusion and functional results supports the validity of the present findings.

## 5. Conclusions

FTD patients with parkinsonism manifest specific putaminal volume reduction as well as reduced structural and functional connectivity between the basal ganglia and SMA. The present findings support the hypothesis of neurodegenerative processes in specific corticobasal ganglia-thalamocortical motor loops as a putative pathophysiological mechanism responsible for parkinsonism in patients with FTD. The present results gain new insights into the pathophysiology of parkinsonism in FTD and provide the theoretical basis for novel neuromodulation strategies.

## Figures and Tables

**Table 1:** Demographic and clinical characteristics of healthy controls and FTD patients with and without parkinsonism (Park+ and Park−).

	HC (N = 30)	FTD (N = 30)	p *	Park− (N = 18)	Park+ (N = 12)	p *
**Demographic/clinical features**						
Age	68.2 ± 6.4	70.1 ± 7.4	ns	68.1 ± 7.8	73.3 ± 5.8	ns
Female/male, *n*	9/21	9/21	ns	7/11	2/10	ns
Disease duration, y	-	3.8 ± 1.9	-	3.7 ± 1.9	4.1 ± 1.8	ns
FTD-subtype (nfv-PPA/bv-FTD), *n*	-	19/11	-	10/8	9/3	ns
**Neuropsychological scores**						
CDR-FTD	-	6.4 ± 3.4	-	6.5 ± 3.3	6.3 ± 3.7	ns
MMSE	-	20.6 ± 7.1	-	21.2 ± 7.3	19.7 ± 7.1	ns
FAB	-	10.8 ± 4.3	-	11.7 ± 4.1	9.2 ± 4.2	ns
TMT A, sec	-	142.3 ± 99.1	-	135.5 ± 104.8	155.5 ± 91.7	ns
TMT B, sec	-	224.5 ± 90.5	-	196.7 ± 96.9	276.9 ± 46.0	ns
VSF	-	20.4 ± 11.4	-	21.3 ± 11.8	18.8 ± 10.9	ns
VPF	-	14.0 ± 12.1	-	14.9 ± 13.0	12.1 ± 10.6	ns
MDS-UPDRS-III §	-	17.8 ± 10.4 §	-	-	17.8 ± 10.4	-

Values are reported as mean ± standard deviation; *n*, number; y, years; ns, not statistically significant. FTD: frontotemporal degeneration; HC: healthy controls; bv-FTD: behavioral variant, frontotemporal degeneration; nfv-PPA: non-fluent variant, primary progressive aphasia; CDR-FTD: Clinical Dementia Rating scale & Frontotemporal Dementia; MMSE: Mini-Mental State Examination; FAB: frontal assessment battery; TMT A/B: trail-making test A/B; VSF: verbal semantic fluency test; VPF: verbal phonemic fluency test; MDS-UPDRS-III: Movement Disorder Society-sponsored revision of the Unified Parkinson’s Disease Rating Scale, part III. § in Park+ FTD patients. p * Mann–Whitney U-test and Chi-square test for continuous and dichotomous variables, respectively (*p* < 0.05).

**Table 2:** Global thickness and basal ganglia and thalamic volumes in healthy controls and FTD patients with and without parkinsonism (Park+ and Park−).

	HC	Park−	Park+	p *	H	Post hoc
L global cortical thickness (mm)	2.361 ± 0.08	2.217 ± 0.12	2.204 ± 0.12	**<0.001**	21.02	HC-Park− ***p* < 0.001**
						HC-Park+ ***p* = 0.001**
						Park−Park+ ns
R global cortical thickness (mm)	2.356 ± 0.08	2.279 ± 0.11	2.252 ± 0.09	**0.003**	10.95	HC-Park− ***p* = 0.038**
						HC-Park+ ***p* = 0.007**
						Park−Park+ ns
L global cortical volume	0.1344 ± 0.0093	0.1195 ± 0.0146	0.1197 ± 0.0167	**<0.001**	17.07	HC-Park− ***p* = 0.001**
						HC-Park+ ***p* = 0.009**
						Park−Park+ ns
R global cortical volume	0.1377 ± 0.0079	0.1278 ± 0.0123	0.1261 ± 0.0140	**<0.001**	13.01	HC-Park− ***p* = 0.006**
						HC-Park+ ***p* = 0.016**
						Park− Park+ ns
L putamen, fraction	0.0028 ± 0.0003	0.0025 ± 0.0006	0.0023 ± 0.0005	**0.010**	9.22	HC-Park− ns
						HC-Park+ ***p* = 0.016**
						Park− Park+ ns
R putamen, fraction	0.0029 ± 0.0002	0.0027 ± 0.0004	0.0025 ± 0.0004	**0.004**	10.86	HC-Park− ns
						HC-Park+ ***p* = 0.004**
						Park− Park+ ns
L globus pallidus, fraction	0.0012 ± 0.0001	0.0011 ± 0.0002	0.0010 ± 0.0002	ns	-	-
R globus pallidus, fraction	0.0012 ± 0.0002	0.0011 ± 0.0001	0.0011 ± 0.0002	ns	-	-
L thalamic fraction, fraction	0.0046 ± 0.0005	0.0041 ± 0.0005	0.0041 ± 0.0005	**<0.001**	17.10	HC-Park− ***p* = 0.002**
						HC-Park+ ***p* = 0.003**
						Park−Park+ ns
R thalamic fraction, fraction	0.0045 ± 0.0003	0.0043 ± 0.0005	0.0041 ± 0.0004	**0.009**	9.33	HC-Park− ns
						HC-Park+ ***p* = 0.007**
						Park− Park+ ns

* Differences between groups were assessed using the Kruskal–Wallis test, Bonferroni-corrected for multiple comparisons (mean ± standard deviation, *p*, H, and post hoc test are displayed). HC = healthy controls; L = left; R = right; ns = not statistically significant; SD = standard deviation. Raw cortical, putaminal, pallidal, and thalamic volumes were normalized within each subject as a ratio of intracranial volume and reported as a fraction.

**Table 3:** FA values of the SMA-subcortical and M1-subcortical WM tracts and RSFC values of SMA-subcortical and M1-subcortical ROI pairs in healthy controls and FTD patients with and without parkinsonism (Park+ and Park−).

	HC	Park−	Park+	p *	H	Post Hoc
**WM Tracts–FA**						
SMA-putamen	0.478 ± 0.04	0.454 ± 0.05	0.433 ± 0.03	**0.003**	11.31	HC-Park− ns
						HC-Park+ ***p* = 0.003**
						Park–Park+ ns
SMA-pallidus	0.500 ± 0.04	0.481 ± 0.05	0.455 ± 0.02	**0.006**	10.36	HC-Park− ns
						HC-Park+ ***p* = 0.004**
						Park–Park+ ns
SMA-thalamus	0.504 ± 0.04	0.487 ± 0.05	0.462 ± 0.03	**0.012**	8.93	HC-Park− ns
						HC-Park+ ***p* = 0.008**
						Park–Park+ ns
M1-putamen	0.496 ± 0.05	0.472 ± 0.05	0.452 ± 0.03	ns	-	-
M1-pallidus	0.518 ± 0.05	0.497 ± 0.05	0.478 ± 0.03	ns	-	-
M1-thalamus	0.519 ± 0.05	0.510 ± 0.05	0.492 ± 0.04	ns	-	-
**ROI pairs RSFC–r (Z-transformed)**						
SMA-putamen	0.689 ± 0.25	0.714 ± 0.21	0.465 ± 0.19	**0.012**	8.76	HC-Park− ns
						HC-Park+ ***p* = 0.017**
						Park—Park+ ***p* = 0.027**
SMA-pallidus	0.492 ± 0.19	0.485 ± 0.17	0.346 ± 0.19	ns	-	-
SMA-thalamus	0.665 ± 0.24	0.793 ± 0.29	0.604 ± 0.36	ns	-	-
M1-putamen	0.638 ± 0.29	0.698 ± 0.22	0.510 ± 0.33	ns	-	-
M1-pallidus	0.465 ± 0.23	0.487 ± 0.16	0.348 ± 0.21	ns	-	-
M1-thalamus	0.717 ± 0.31	0.828 ± 0.31	0.626 ± 0.36	ns	-	-

* Differences between groups were assessed using the Kruskal–Wallis test, Bonferroni-corrected for multiple comparisons (mean ± standard deviation, *p*, H, and post hoc test are displayed). FA = fractional anisotropy; FTD = frontotemporal degeneration; HC = healthy controls; M1 = primary motor cortex; ns = not statistically significant; ROI = region of interest; RSFC = resting-state functional connectivity; SD= standard deviation; SMA = supplementary motor area; WM = white matter.

## Data Availability

The datasets presented in this article are not readily available because of patient confidentiality and participant privacy restrictions. Requests to access the datasets should be directed to the corresponding author.

## Supplementary Materials

The following supporting information can be downloaded at: https://www.mdpi.com/article/10.3390/biomedicines11020522/s1, Table S1: Cortical regions displaying significant thinning in Park+ and Park− patients respect to healthy controls.
