# Supplementary material for: Parkinsonism Is Associated with Altered SMA-Basal Ganglia Structural and Functional Connectivity in Frontotemporal Degeneration
Source: Biomedicines. 2023 Feb 10;11(2):522. doi: 10.3390/biomedicines11020522 (PMC9953061; doi:10.3390/biomedicines11020522)
Supplement: Supplementary file 1 [file biomedicines-11-00522-s001.zip › biomedicines-2212793-supplementary.pdf]

Supplementary Materials.

Parkinsonism is Associated with Altered SMA-Basal Ganglia Structural and Functional Connectivity in Frontotemporal Degeneration

MRI analysis

Data preprocessing

Anatomical preprocessing was performed using *fMRIPrep* 20.1.1([1,2]; RRID:SCR_016216), which is based on *Nipype* 1.5.0 ([3,4]; RRID:SCR_002502). Each of the T1-weighted (T1w) images were preprocessed with the following pipeline: First, the T1w image was corrected for intensity non-uniformity (INU) with N4BiasFieldCorrection [5], distributed with ANTs 2.2.0 ([6], RRID:SCR_004757), and used as T1w-reference throughout the workflow. The T1w-reference was then skull-stripped with a *Nipype* implementation of the antsBrainExtraction.sh workflow (from ANTs), using OASIS30ANTs as target template. Brain tissue segmentation of cerebrospinal fluid (CSF), white matter (WM) and gray-matter (GM) was performed on the brain-extracted T1w using fast (FSL 5.0.9, RRID:SCR_002823, [7]). Brain surfaces were reconstructed using recon-all (FreeSurfer 6.0.1, RRID:SCR_001847, [8]), and the brain mask estimated previously was refined with a custom variation of the method to reconcile ANTs-derived and FreeSurfer-derived segmentations of the cortical gray-matter of Mindboggle (RRID:SCR_002438, [9]). Volume-based spatial normalization to two standard spaces (MNI152NLin2009cAsym, MNI152NLin6Asym) was performed through nonlinear registration with antsRegistration (ANTs 2.2.0), using brain-extracted versions of both T1w reference and the T1w template. The following templates were selected for spatial normalization: *ICBM 152 Nonlinear Asymmetrical template version 2009c* [10], RRID:SCR_008796; TemplateFlow ID: MNI152NLin2009cAsym), *FSL's MNI ICBM 152 non-linear 6th Generation*

Asymmetric Average Brain Stereotaxic Registration Model ([11], RRID:SCR_002823; TemplateFlow ID: MNI152NLin6Asym].

Diffusion data were visually inspected for artifacts and preprocessed using different tools from FDT (FMRIB Diffusion Toolbox, part of FSL (FMRIB's Software Library v.6.0.4, http://www.fmrib.ox.ac.uk/fsl/; [12]). Images were corrected for eddy current distortion and head motion using a 12-parameter affine registration to the first no-diffusion weighted volume of each subject, and the gradient directions were rotated accordingly [13]. Non-brain tissue was removed from the eddy-corrected images using the Brain Extraction Tool (BET; [14]) creating a binary mask of the brain. Then, fractional anisotropy (FA) maps were estimated at the individual level using the DTIFIT tool by fitting a tensor model to the eddy-corrected and brain masked diffusion data. Registration between diffusion, structural, and standard space images was performed within FDT. Transformation matrices, and their inverses, were created to transform images between spaces.

Functional preprocessing was also performed using *fMRIPrep* 20.1.1. For each subject, the following preprocessing was performed: first, a reference volume and its skull-stripped version were generated using a custom methodology of *fMRIPrep*. Head-motion parameters with respect to the BOLD reference (transformation matrices, and six corresponding rotation and translation parameters) are estimated before any spatiotemporal filtering using mcflirt (FSL 5.0.9, [15]). BOLD runs were slice-time corrected using 3dTshift from AFNI 20160207 ([16], RRID:SCR_005927). Susceptibility distortion correction (SDC) was omitted. The BOLD reference was then co-registered to the T1w reference using bbregister (FreeSurfer) which implements boundary-based registration [17]. Co-registration was configured with six degrees of freedom. The BOLD time-series (including slice-timing correction when applied) were resampled onto their original, native space by applying the transforms to correct for head-motion. These resampled BOLD time-series will be referred to as preprocessed BOLD in original space, or just preprocessed BOLD. The BOLD time-series were

resampled into standard space, generating a preprocessed BOLD run in MNI152NLin2009cAsym space. First, a reference volume and its skull-stripped version were generated using a custom methodology of fMRIPrep. Automatic removal of motion artifacts using independent component analysis (ICA-AROMA, [18]) was performed on the preprocessed BOLD on MNI space time-series after removal of non-steady state volumes and spatial smoothing with an isotropic, Gaussian kernel of 6mm FWHM (full-width half-maximum). Corresponding "non-aggressively" denoised runs were produced after such smoothing. Functional preprocessed data were finally subjected to WM and CSF signal regression and high-pass filtering (100-seconds cut-off) [18].

Supplementary Table S1. Cortical regions displaying significant thinning in Park+ and Park− respect to healthy controls (HC). The table reports the list of the clusters (each raw represents one cluster) along with the maximum value found in the cluster (Max), the vertex at which this maximum value was found (Vertex Max), the surface area of the cluster (Size) and the Talairach coordinates of the maximum and corresponding region labeling. All results presented at the corrected threshold ($p < 0.05$).

Contrast	Max -log10(pvalue)	Vertex Max	Size (mm^2)	Talairach coordinates			Region
				x	y	z	
**HC-Park+**	6.981	134025	4763.75	-12.6	43.0	14.5	Left superior frontal gyrus
	5.338	93461	2556.89	-42.0	-2.8	-30.0	Left inferior temporal gyrus
	4.311	47417	1447.07	-42.7	36.7	-13.5	Left inferior frontal gyrus pars orbitalis
	4.150	114295	943.29	-54.5	-51.8	-13.4	Left inferior temporal gyrus
	4.186	49891	791.56	-34.6	33.0	25.3	Left rostral middle frontal gyrus
	4.849	59336	742.77	-52.9	5.8	26.8	Left precentral
**HC-Park−**							
	7.620	18177	11866.56	-37.2	49.8	-1.0	Left rostral middle frontal
	6.969	62700	3131.53	-47.1	-14.4	-31.5	Left inferior temporal
	5.476	103901	1866.67	-41.3	9.1	18.3	Left inferior frontal gyrus, pars opercularis
	6.227	126795	1178.10	-15.0	-46.3	36.4	Left precuneus
	4.202	93275	669.96	-20.8	-58.9	22.4	Left precuneus
	4.865	9533	554.42	-23.4	-60.8	29.6	Left superior parietal gyrus
	3.400	163011	469.79	-33.2	-48.4	-6.5	Left fusiform
	5.505	97429	2430.68	45.6	-15.3	-28.1	Right inferior temporal
	4.646	149261	2054.59	13.6	27.1	27.5	Right superior frontal
	5.528	116267	879.15	22.8	46.7	18.6	Right rostral middle frontal

References

1. Esteban, O.; Markiewicz, C.J.; Blair, R.W.; Moodie, C.A.; Isik, A.I.; Erramuzpe, A.; Kent, J.D.; Goncalves, M.; DuPre, E.; Snyder, M.; et al. FMRIPrep: A Robust Preprocessing Pipeline for Functional MRI. *Nature Methods* **2019**, *16*, 111–116, doi:10.1038/s41592-018-0235-4.
2. Esteban, O. FMRIPrep: A Robust Preprocessing Pipeline for Functional MRI (Version 20.1.1). *Zenodo* **2020**, doi:http://doi.org/10.5281/zenodo.3876458.
3. Gorgolewski, K.; Burns, C.D.; Madison, C.; Clark, D.; Halchenko, Y.O.; Waskom, M.L.; Ghosh, S.S. Nipype: A Flexible, Lightweight and Extensible Neuroimaging Data Processing Framework in Python. *Front. Neuroinform.* **2011**, *5*, doi:10.3389/fninf.2011.00013.
4. Gorgolewski, K. "Nipype." Software. *Zenodo* **2018**, doi:https://doi.org/10.5281/zenodo.596855.
5. Tustison, N.J.; Avants, B.B.; Cook, P.A.; Zheng, Y.; Egan, A.; Yushkevich, P.A.; Gee, J.C. N4ITK: Improved N3 Bias Correction. *IEEE Transactions on Medical Imaging* **2010**, *29*, 1310–1320, doi:10.1109/TMI.2010.2046908.
6. Avants, B.B.; Epstein, C.L.; Grossman, M.; Gee, J.C. Symmetric Diffeomorphic Image Registration with Cross-Correlation: Evaluating Automated Labeling of Elderly and Neurodegenerative Brain. *Medical Image Analysis* **2008**, *12*, 26–41, doi:10.1016/j.media.2007.06.004.
7. Zhang, Y.; Brady, M.; Smith, S. Segmentation of Brain MR Images through a Hidden Markov Random Field Model and the Expectation-Maximization Algorithm. *IEEE Transactions on Medical Imaging* **2001**, *20*, 45–57, doi:10.1109/42.906424.
8. Dale, A.M.; Fischl, B.; Sereno, M.I. Cortical Surface-Based Analysis: I. Segmentation and Surface Reconstruction. *NeuroImage* **1999**, *9*, 179–194, doi:10.1006/nimg.1998.0395.
9. Klein, A. Mindboggling Morphometry of Human Brains. **2017**, doi:https://doi.org/10.1371/journal.pcbi.1005350.
10. Fonov, V.; Evans, A.; McKinstry, R.; Almli, C.; Collins, D. Unbiased Nonlinear Average Age-Appropriate Brain Templates from Birth to Adulthood. *NeuroImage* **2009**, *47*, S102, doi:10.1016/S1053-8119(09)70884-5.
11. Evans, A.C.; Janke, A.L.; Collins, D.L.; Baillet, S. Brain Templates and Atlases. *NeuroImage* **2012**, *62*, 911–922, doi:10.1016/j.neuroimage.2012.01.024.
12. Smith, S.M.; Jenkinson, M.; Woolrich, M.W.; Beckmann, C.F.; Behrens, T.E.J.; Johansen-Berg, H.; Bannister, P.R.; De Luca, M.; Drobnjak, I.; Flitney, D.E.; et al. Advances in Functional and Structural MR Image Analysis and Implementation as FSL. *NeuroImage* **2004**, *23*, S208–S219, doi:10.1016/j.neuroimage.2004.07.051.
13. Leemans, A.; Jones, D.K. The B-Matrix Must Be Rotated When Correcting for Subject Motion in DTI Data. *Magn Reson Med* **2009**, *61*, 1336–1349, doi:10.1002/mrm.21890.
14. Smith, S.M. Fast Robust Automated Brain Extraction. *Human Brain Mapping* **2002**, *17*, 143–155, doi:10.1002/hbm.10062.
15. Jenkinson, M.; Bannister, P.; Brady, M.; Smith, S. Improved Optimization for the Robust and Accurate Linear Registration and Motion Correction of Brain Images. *Neuroimage* **2002**, *17*, 825–841.
16. Cox, R.W.; Hyde, J.S. Software Tools for Analysis and Visualization of FMRI Data. *NMR Biomed* **1997**, *10*, 171–178, doi:10.1002/(sici)1099-1492(199706/08)10:4/5<171::aid-nbm453>3.0.co;2-l.

17. Greve, D.N.; Fischl, B. Accurate and Robust Brain Image Alignment Using Boundary-Based Registration. *Neuroimage* **2009**, *48*, 63–72, doi:10.1016/j.neuroimage.2009.06.060.
18. Pruim, R.H.R.; Mennes, M.; van Rooij, D.; Llera, A.; Buitelaar, J.K.; Beckmann, C.F. ICA-AROMA: A Robust ICA-Based Strategy for Removing Motion Artifacts from FMRI Data. *Neuroimage* **2015**, *112*, 267–277, doi:10.1016/j.neuroimage.2015.02.064.
